# Curcuminoids as EBV Lytic Activators for Adjuvant Treatment in EBV-Positive Carcinomas

**DOI:** 10.3390/cancers10040089

**Published:** 2018-03-22

**Authors:** Octavia Ramayanti, Mitch Brinkkemper, Sandra A. W. M. Verkuijlen, Leni Ritmaleni, Mei Lin Go, Jaap M. Middeldorp

**Affiliations:** 1 Department of Pathology, VU University Medical Center, 1081HV Amsterdam, The Netherlands; octavia.rm13@gmail.com (O.R.); mitch.brinkkemper@gmail.com (M.B.); s.verkuijlen@vumc.nl (S.A.W.M.V.); 2 Laboratory of Medicinal Chemistry, Gadjah Mada University, Yogyakarta 55281, Indonesia; ritmaleni@ymail.com; 3 Department of Pharmacy, National University of Singapore, Singapore 117543, Singapore; meilin.go@nus.edu.sg

**Keywords:** Epstein-Barr virus, EBV, nasopharyngeal carcinoma, gastric cancer, curcumin, curcuminoids, virus-targeted therapy, cytolytic virus activation (CLVA), oral adjuvant therapy

## Abstract

Epstein-Barr virus (EBV) persists in nasopharyngeal (NPC) and gastric carcinomas (EBVaGC) in a tightly latent form. Cytolytic virus activation (CLVA) therapy employs gemcitabine and valproic acid (GCb+VPA) to reactivate latent EBV into the lytic phase and antiviral valganciclovir to enhance cell death and prevent virus production. CLVA treatment has proven safe in phase-I/II trials with promising clinical responses in patients with recurrent NPC. However, a major challenge is to maximize EBV lytic reactivation by CLVA. Curcumin, a dietary spice used in Asian countries, is known for its antitumor property and therapeutic potential. Novel curcuminoids that were developed to increase efficacy and bioavailability may serve as oral CLVA adjuvants. We investigated the potential of curcumin and its analogs (curcuminoids) to trigger the EBV lytic cycle in EBVaGC and NPC cells. EBV-reactivating effects were measured by immunoblot and immunofluorescence using monoclonal antibodies specific for EBV lytic proteins. Two of the hit compounds (**41**, **EF24**) with high lytic inducing activity were further studied for their synergistic or antagonistic effects when combined with GCb+VPA and analyzed by cytotoxicity and mRNA profiling assays to measure the EBV reactivation. Curcuminoid as a single agent significantly induced EBV reactivation in recombinant GC and NPC lines. The drug effects were dose- and time-dependent. Micromolar concentration of curcuminoid **EF24** enhanced the CLVA effect in all cell systems except SNU719, a naturally infected EBVaGC cell that carries a more tightly latent viral genome. These findings indicated that **EF24** has potential as EBV lytic activator and may serve as an adjuvant in CLVA treatment.

## 1. Introduction

Epithelial malignancies associated with Epstein-Barr virus (EBV) infection include undifferentiated nasopharyngeal carcinoma (NPC) and a subset of gastric carcinoma (GC) [1]. EBV is detected in most of NPC cases in endemic countries. More than 80% of post-surgical gastric stump or remnants, the lymphoepithelioma-like (LEL) GC, and about 10% of gastric adenocarcinoma cases around the world show evidence of EBV infection called EBV associated gastric carcinoma (EBVaGC). Overall, patients with EBVaGC had longer survival than those GC patients with no evidence of EBV [2,3]. In NPC, both hyperplastic, pre-invasive, and invasive lesions of the nasopharynx show the presence of monoclonal viral episomes [1], whereas EBV genomes persist only in fully malignant EBVaGC lesions in the proximal stomach [2,3]. Contrary to EBVaGC, which occurs worldwide, NPC is rare in most parts of the world, but notoriously common in the Southern China, Southeast Asia, North America, and the Mediterranean region [1,2].

Most EBV-associated carcinomas contain viral DNA that persists in a latent state with only a limited set of viral genes being expressed. EBV nuclear antigen 1 (EBNA1), small RNAs EBER1 and 2, BARF1 protein, and BamHI-A rightward transcripts (BART) encoding 40 miRNAs are expressed in all carcinoma cells [1]. EBVaGC, however, has a unique modified latency type I expression pattern with a variable expression of LMP2A, but no LMP1. In contrast, 50–80% of NPC cases express LMP1 in addition to LMP2A, which characterizes the NPC latency pattern as type II [2].

Besides personalized T-cell based immunotherapy, to date, no effective virus-targeted treatment has been developed for NPC and EBVaGC [4,5]. Recently, chemical EBV reactivation followed by administration of an antiviral cytolytic drug was proposed for the treatment of EBV-positive lymphomas and carcinomas refractory to conventional chemotherapy or radiation [4,5,6,7]. A recent clinical trial on cytolytic virus activation (CLVA) therapy using a combination of gemcitabine (GCb), valproic acid (VPA) and ganciclovir (GCV) demonstrated virus reactivation in vivo associating with promising clinical responses in end-stage NPC patients [6,7]. To ensure the selective killing of EBV-positive tumors, effective initiation of EBV lytic cycle and expression of viral kinases are required. These kinases phosphorylate nucleoside analogs (e.g., acyclovir, (val)ganciclovir; GCV) into their active forms which cause DNA chain termination leading to lysis of tumor cells and curtailing the release of infectious viral particles [4,8].

The mechanisms of induction of EBV lytic replication from latency using chemical inducers such as histone deacetylase inhibitors (HDACi), DNA demethylating agents, aspirin, NF-κB inhibitory compounds and chemotherapeutic drugs that induce DNA-damage repair have been extensively investigated and recently reviewed in detail [4,8,9]. Induction of host cell differentiation and inhibition of NF-κB are common themes that trigger EBV reactivation in latently infected cells [8,9,10]. Most existing chemical activators of EBV lytic cycle thus far tested in clinical trials are associated with significant toxicities and restricted bioavailability [8,9]. Recent studies have identified various novel small organic compounds for potential EBV lytic inducers [9,10,11,12]. Whether these compounds will be tolerated in clinical settings remains to be determined.

Meanwhile, several human clinical trials have reported that curcumin, a polyphenolic compound known derived from *Curcuma longa* possess various therapeutic properties including anti-oxidant, analgesic, anti-inflammatory and anti-cancer activities due to its effect on multiple biological pathways including the inhibition of NF-κB [9,13,14]. Importantly, curcumin is “generally recognized as safe” by the U.S. Food and Drug Administration, and is being used as adjuvant in approved clinical cancer therapies [13,14]. Curcumin and its derivatives (known as curcuminoids) used alone or in combination with other drugs, increase cell death by modulating Cox-2 and NF-κB pathways in a wide variety of tumor cells with minimal cytotoxicity [13,14,15]. Several curcuminoids have been developed to improve the known pharmacokinetic limitations (poor oral bioavailability, rapid metabolism) of curcumin [16,17,18,19,20,21,22,23]. Curcumin and novel curcuminoids have recently been shown to limit the growth of NPC and GC cells in vitro and in a mouse tumor model, but without addressing the role of EBV in these tumors [14,16,21,22,23].

**Figure 1:**
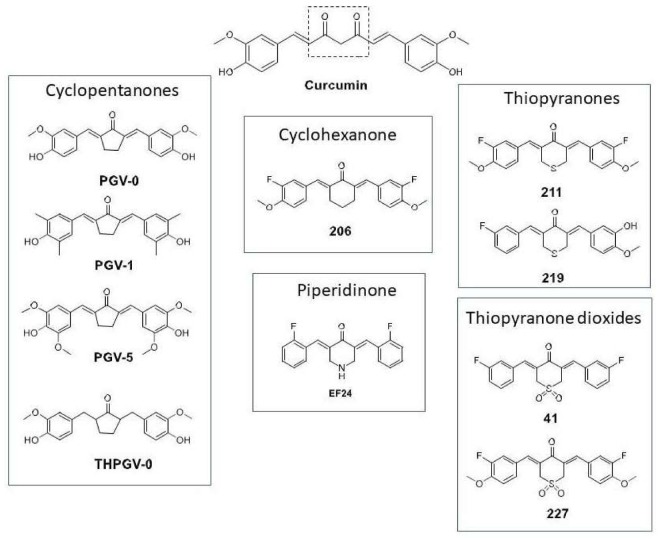
Novel curcuminoids through structural modification of curcumin to improve uptake. Curcumin structure and modifications of curcumin at its β-diketone linker and terminal phenyl rings to improve stability, bioavailability and pharmacokinetic profile as described in the Materials and Methods section.

The central conjugated β-diketone linker in curcumin has been identified to contribute to its chemical and metabolic instability [18]. Replacing the conjugated linker with a monocarbonyl cross-conjugated dienone that is embedded within a ring structure has been widely employed as a stabilizing modification. In this report, we explored various structural curcuminoid types that embodied this modification [17,18]. Curcuminoids with five different ring structures were investigated [17,18,19,20], namely cyclopentanones **PGV-0**, **PGV-1**, **PGV-5**, **THPGV-0**, cyclohexanone **206**, piperidinone **EF24**, thiopyranones **211**, **219** and thiopyranone dioxides **41**, **227** (Figure 1).

The cyclopentanones were obtained from the UGM-VU collection of curcuminoids and two members (**PGV-0**, **PGV-1**) have been reported to possess cytotoxic, antiproliferative and anti-angiogenesis properties in tumor cells by inhibiting COX-2 and NF-κB signaling [19,20]. The piperidinone **EF24**, a widely investigated curcuminoid with improved stability and bioavailability, has pleiotropic effects on inflammatory and oncogenic signaling pathways [21,22,23]. In particular, EF24 has strong inhibitory effects on IKK, thus inhibiting NF-κB nuclear translocation and blocking NF-κB driven transcriptional activation [22,23]. Like the cyclohexanones, thiopyranones and thiopyranone dioxides, **EF24** induced apoptosis in leukemic cells [17]. They were also more potent than curcumin, with the exception of the cyclohexanone **206** and thiopyranone **211** [18]. The most potent analogs were **41** > **227** > **EF24**, based on cell-based growth inhibitory concentrations (IC_50_). The apoptotic effects of **41** and **227** were attributed to activation of the unfolded protein response in response to heightened endoplasmic reticulum (ER) stress induced by these compounds [18].

It is reported that reactivation of the latent viral genome in EBV associated cancers can cause cancer cell death [10,24,25,26]. Due to the need for a highly efficacious EBV targeted therapy with lower toxicity and preferably oral drug availability, a detailed investigation into the potential of curcuminoids for initiating EBV reactivation in the context of CLVA therapy is needed. Here, we screen and identify the EBV lytic induction potential of curcuminoids used as a single agent or as an adjuvant to CLVA therapy in EBV-associated carcinoma cells. Considering the relevance of cellular background for EBV lytic reactivation, we confirm the cells’ ability to express EBV lytic genes in multiple NPC and EBVaGC cell lines, carrying either a recombinant EBV genome (HONE-EBV and AGS-BX1 model systems) or a natural EBV genome (C666.1 and SNU-719 human tumor-derived cell lines). These curcuminoids are structurally distinct (Figure 1) and synergize with CLVA regimen to activate the lytic life cycle in latently infected cells while maintaining low toxicity.

## 2. Materials and Methods

### 2.1. Cell Lines

EBV-positive GC cell lines (AGS-BX1, SNU-719) and EBV-positive NPC cell lines (HONE1-EBV, C666.1) were used in this study. Natural EBV genome-carrying SNU-719 cells (purchased from the Korean Cell Line Bank, Seoul, Korea), natural EBV genome-carrying C666.1 NPC cells and recombinant AGS-BX1 cells were cultured as described before [6,12,24,27]. AGS-BX1 harbors a recombinant EBV genome with an insertion of the neomycin-resistance gene and the green fluorescent protein (GFP) gene that disrupts the TK gene (kindly provided by L. Hutt-Fletcher, Louisiana State University, Schreveport, LA, USA) [6]. HONE1-EBV was generated by introducing a green fluorescent protein (GFP) open reading frame in the recombinant Akata EBV genome into the EBV-negative NPC cell line HONE1 (gift from Dr. Sai Wah Tsao, Hong Kong University, Hong Kong, China) [24]. EBV-negative GC (AGS) and EBV-negative NPC (HONE1) parental cell lines were used as negative controls. AGS, HONE1, and HONE1-EBV cells were maintained as detailed by Hui et al. [24,28].

### 2.2. Chemicals, Plasmids, and Antibodies

Natural purified curcumin was purchased from Sigma (Sigma-Aldrich, St. Louis, MO, USA), whereas novel analogs curcumin compounds were kindly provided by Mei Lin Go at The National University of Singapore (NUS), Singapore and by Ritmaleni at The University of Gadjah Mada (UGM), Indonesia in collaboration with Henk Timmerman of the Vrije Universiteit Amsterdam, The Netherlands (UGM-VU). The chemical structure of curcuminoids is summarized in Figure 1. The NUS collection consists of molecules with replacement of β-diketone by cyclohexanone (compound **206**), thiopyranone (compound **211**, **219**), thiopyranone dioxide (compound **41**, **227**) and piperidinone (**EF24**) moieties [17]. NUS compounds acted as activators of endoplasmic reticulum (ER) stress signaling pathways and apoptotic cell death in leukemic cells [18]. Penta Gama Vunon (PGV) is novel curcuminoid from UGM-VU collection named benzylidenecyclopentanone. In PGV, the methylene and carbonyl groups have been omitted to produce more stable and potent compounds retaining anti-oxidant and anti-inflammatory activities. Two benzylidenecyclopentanone derivatives (**PGV0** and **PGV1**) demonstrated cytotoxic, anti-proliferative and anti-angiogenesis properties in tumor cells [19,20]. Suberoylanilide hydroxamic acid (SAHA), sodium butyrate (SB), gemcitabine (GCb) and valproic acid (VPA) purchased from Sigma (Sigma-Aldrich, St. Louis, MO, USA) were used as positive control lytic activators [6,9,24]. In all experiments, 1% DMSO in culture medium was used as negative control. Two murine monoclonal antibodies were used for immunofluorescence and immunoblot analysis, i.e., BZ1 antibody for detection of the lytic-switch protein BZLF1/Zebra and OT14E antibody for detection of the BMRF1/EA-D protein [6,24,26,27,28,29]. Rabbit antibodies against active caspase 3 and PARP-1/p89 for detecting apoptosis-related proteins were purchased from Promega (Promega Benelux, Leiden, The Netherlands), respectively.

### 2.3. Cytotoxicity and Cell Viability Assays

The cytotoxicity of hit compounds was evaluated by 3-(4,5-dimethylthiazol-2-yl)-2,5-diphenyl tetrazolium bromide (MTT, Sigma-Aldrich, Zwijndrecht, The Netherlands) proliferation assay. Briefly, EBV-negative and -positive NPC and GC cells (2–5 × 10^4^ cells) were seeded in 96-well culture plates without drug and allowed to adhere for 24 h. Subsequently, *t* = 0 was measured using MTT and hit compounds (**41**, **EF24**) including positive lytic activators SAHA and GCb+VPA were added at different concentrations (in 2- or 5-fold). After 72 h, cells were lysed in DMSO and absorbance was determined at 540 nm in a plate reader [6,27,28]

**Figure 2:**
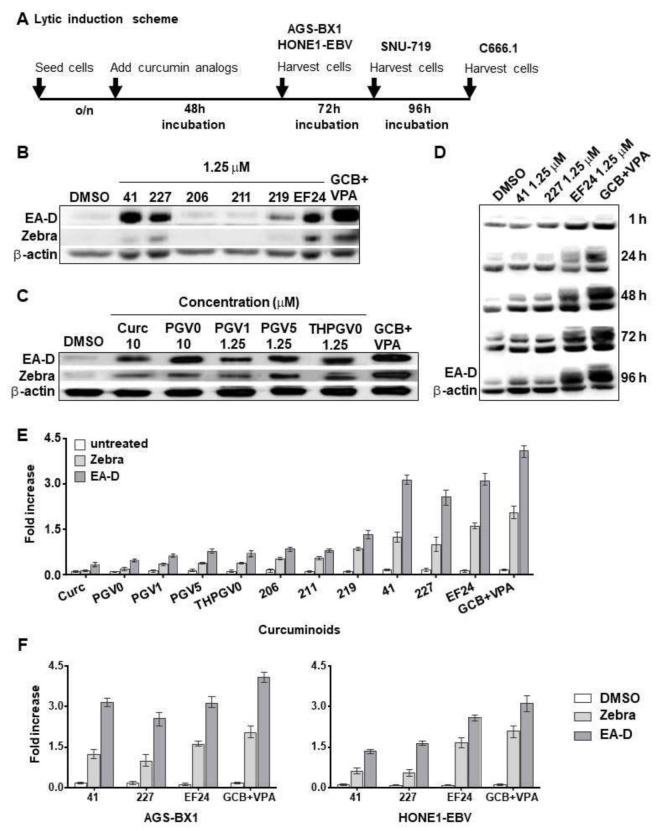
Different lytic induction effects of curcuminoids in gastric (GC) and nasopharyngeal (NPC) carcinoma cell lines carrying recombinant Epstein-Barr virus (EBV) genomes. Curcumin and curcuminoids promote the accumulation of Zebra and EA-D lytic proteins in gastric (AGS-BX1) and nasopharyngeal (HONE1-EBV) carcinoma cells. (**A**) Experimental scheme representing the curcuminoid treatment of EBV-positive GC and NPC cells; (**B**) Treatment at 1.25 µM concentration resulted in strong lytic induction effects of hit compounds **41**, **227**, and **EF24**; (**C**) Lytic induction effects of curcuminoids in HONE1-EBV cells; (**D**) Expression of Zebra and EA-D lytic proteins in AGS-BX1 cells after treatment with 1.25 µM of hit compounds (**41**, **227**, **EF24**) for 1, 24, 48, 72, or 96 h or no treatment analyzed by immunoblot; (**E**) The relative level of EBV lytic proteins (Zebra, EA-D) induced by curcuminoids was assessed after normalisation with β-actin as loading control. Results are presented as fold increase of EBV lytic proteins of treated cells compared with untreated cells; (**F**) The lytic induction effect of hit compounds (**41**, **227**, **EF24**) is more effective and intense in AGS-BX1 (evidenced by 1.5 to 3-fold increase) compared with that of HONE1-EBV (1.2 to 2-fold increase) cells. AGS-BX1 and HONE1-EBV cells treated with 3 mmol/L GCb and 0.3 mmol/L valproic acid (VPA) were included as positive controls (**B**–**F**).

The synergistic killing effect of hit compounds (**41**, **EF24**) and GCb+VPA was determined by trypan blue exclusion assay. EBV-positive GC and NPC cells grown to 70% confluence were treated with a combination of hit compound (**41**, **EF24**) and GCb+VPA or GCb+VPA alone for 48–96 h depending on cell types (Figure 2A). Treatment with 5 µM SAHA or 3 mM SB was used as positive control [9,24,28]. Results are presented as percentages of viable cell populations among treated cells compared with those of untreated (DMSO) control. Experiments were repeated three times independently.

### 2.4. Immunoblot Analysis

To analyze the expression of EBV lytic proteins, NPC and GC cells were treated with low (10 nm) and high (1.25 µM) concentrations of curcuminoids for 48–96 h (Figure 2A). Treatments with three activators at their lytic concentrations (3 µmol/L GCb + 0.3 mmol/L VPA, 5 µM SAHA, 30 mM SB) were included in the immunoblot analysis [6,24,28]. After treatment, the cells were pelleted and washed once with PBS. Proteins from the cell pellets were extracted and immunoblot analysis to detect Zebra and EA-D lytic proteins were performed as described previously [6,24,29,30].

### 2.5. FACS Analysis, Immunofluorescence Assay and Measurement of Percentage of Cells Induced into Lytic Cycle

FACS analysis was used to screen lytic inducing capacity of various curcuminoids at nanomolar concentrations. GFP intensity representing induced lytic cells was measured by FACSCalibur Flow Cytometer (BD Biosciences, Vianen, The Netherlands). A gate was set to determine the percentage of lytic cells by analyzing the GFP intensity of uninduced AGS-BX1 cells and after the CLVA treatment (GCb+VPA). Expression of GFP in the infected AGS-BX1 and HONE1-EBV cells following curcuminoid treatments visualized and counted under a fluorescence microscope (Leica, Amsterdam, The Netherlands).

Immunofluorescence staining was performed to analyze EBV reactivation in naturally infected GC (SNU-719) and NPC (C666.1) lines [6,24,29,31]. Cells grown on coverslips coated with 0.1% gelatin were treated with either 1.25 µM hit compounds for 72–96 h. Treatment with SAHA and GCb+VPA were included as controls. Cells were fixed with cold methanol-acetone for 10 min and dried at room temperature. Subsequently, nonspecific binding was blocked by incubation in PBS containing 1% FCS. Glass slides were incubated with either Zebra (1:100) or EA-D (1:1000) antibodies in PBS-1%FCS for 60 min at room temperature. After washing with PBS containing 0.05% Tween-20, incubated with the secondary antibody conjugated with fluorescein isothiocyanate (FITC) for 30 min, washed again and slides were mounted in Vectashield (Vector Lab Inc., Peterborough, UK) containing 0.3 µmol/L 4′,6-diamidino-2-phenylindole (DAPI, Roche, Mannheim, Germany). Incubation with PBS-1%FCS but no primary antibody and then with secondary antibody was used as a negative control. Staining patterns were observed under a fluorescence microscope. To quantify GFP-reactive AGS-BX1 and HONE1-EBV and FITC-positive C666.1 and SNU-719 cells, the percentage of positive cells was estimated by counting at least 500 cells from 5 high power fields. Amount of positive populations of AGS-BX1 and HONE1-EBV cells were then subtracted from those of (GFP-positive) untreated (DMSO) control.

### 2.6. Quantitative RT-PCR Assay for the Detection of Specific mRNA Related to EBV Lytic Reactivation

EBV latent and lytic gene expression was quantified by a multiplexed RT-qPCR method using a plasmid pool representing each EBV lytic target gene as described in detail recently [30,32]. EBV-positive GC (AGS-BX1, SNU-719) and NPC (HONE1-EBV, C666.1) cells were treated with a combination of hit compounds (**41**, **EF24**) and GCb+VPA or GCb+VPA alone for 48–96 h. Treatment with SAHA and SB were included in the analysis. After treatment, the cells were pelleted, washed once with PBS and RNA were extracted using Trizol reagent (Invitrogen Life Science, Carlsbad, CA, USA). EBV mRNA lytic gene expression was compared to untreated (DMSO) controls and presented as fold increase. Data were determined from three independent experiments.

## 3. Results

### 3.1. Comparison of Curcumin and Curcuminoids as EBV Lytic Inducer Agents

We have previously reported that the CLVA drug regimen (combined use of GCb and VPA; an FDA approved nucleoside analog and HDAC inhibitor), significantly and synergistically reactivates the latent virus in NPC tumor cells into the lytic replicative phase both in vitro and in vivo and applied this strategy in a clinical Phase-I/-II trial with promising clinical responses [5,6,7]. We hypothesized that curcuminoids may act as EBV lytic induction sensitizer through their NF-κB modulating effect [10,13,14,20,21,22,23]. It was considered that by administering a curcuminoid agent as a potential oral adjuvant to the CLVA regimen a higher percentage of infected cells would enter into lytic phase, thereby improving treatment efficacy.

Here, these structurally diverse curcuminoids were explored for their reactivating effects on the EBV lytic cycle. To this end, we carried out a dose-dependent screen for EBV reactivation (drug concentrations ranging 10 nM to 10 µM) in GFP-modified AGS-BX1 cells carrying recombinant EBV with GFP under lytic (BXLF1) promotor control and measured the green fluorescent intensity of lytic cycle induction by FACS. This cell line is frequently used by others in studies analyzing EBV lytic activation in an epithelial cell background [6,9,12,28]. Initial screening in AGS-BX1 cells revealed that nanomolar concentrations of curcuminoids containing thiopyranone and piperidinone linkers (**211**, **219**, **41**, **227**, **EF24**) gave weak lytic induction signals (Supplementary Materials Figure S1). However compound **41**, **227** and **EF24** demonstrated strong Zebra and EA-D reactivation at 1.25 µM concentration (Figure 2B). Curcumin and **PGV0** stimulated lytic activity at a higher dose (10 µM) (Figure 2C). PGVs containing cyclopentanone linkers (**PGV1**, **PGV5**, **THPGV0**) showed EBV Zebra and EA-D activation with 1.25 µM being the minimal dose for lytic reactivation in HONE1-EBV cells (Figure 2C). To confirm curcuminoid induced reactivation of EBV lytic cycle, we also examined the expression of Zebra (immediate-early) and EA-D (early) proteins in both GC and NPC cells containing native EBV genomes (SNU-719 and C666.1). The experimental scheme to investigate compound induced lytic induction in NPC and GC cells is presented in Figure 2A. This initial assessment led to the selection of three most effective hit compounds, namely the thiopyranone dioxides **41**, **227** and the piperidinone **EF24**, for further evaluation.

### 3.2. Different Lytic Induction Effect of Curcuminoids in Gastric and Nasopharyngeal Carcinoma Cell Line Carrying a Recombinant EBV Genome

Two recombinant cell lines AGS-BX1 and HONE1-EBV, generated by infecting AGS (GC) and HONE1 (NPC) cells with recombinant EBV genome are widely used to study induction of the EBV lytic cycle in vitro [10,12,28]. We observed that incubation of AGS-BX1 cells with hit compounds at 1.25 µM caused a time-dependent increased EA-D activation up to 48 h then stabilized at 72 h (Figure 2D). Strong visible EA-D band produced by AGS-BX1 induced with **EF24** for 48 h revealed that **EF24** is more potent than **41** and **227** (Figure 2D). These data indicated that the lytic induction kinetics varied between curcuminoids, the best and fastest of which was **EF24**. In agreement with a previous study [24], we observed similar kinetics of expression of EBV lytic proteins in NPC cells containing recombinant EBV, HONE1-EBV (data not shown). These data showed that 48 h treatment time is sufficient for EBV lytic reactivation in carcinoma cells carrying recombinant EBV genomes (Figure 2A).

Figure 2E shows a quantitative analysis demonstrating that lytic induction varied among curcumin analogs with different linker modifications. The immunoblot results also indicated that the standard CLVA regimen (GCb+VPA) provides more potent lytic induction than single curcuminoids alone (Figure 2E). We observed that at 1.25 µM concentration of each hit compound was sufficient to induce high expression of EBV immediate-early (Zebra) and early-(EA-D) proteins in both AGS-BX1 and HONE1-EBV (Figure 2E). In AGS-BX1 cells the three hit compounds (**41**, **227**, **EF24**) induced the expression of Zebra and EA-D proteins 1.5–2 fold higher than in HONE1-EBV (Figure 2E; left and right panels).

**Figure 3:**
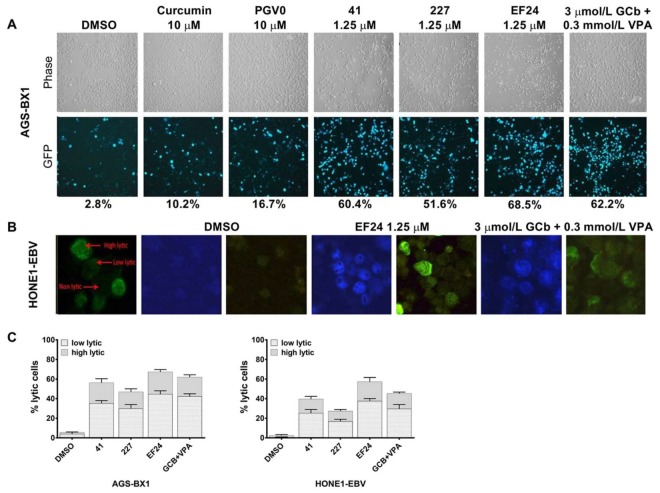
Percentage of AGS-BX1 and HONE1-EBV cells expressing EBV lytic reactivation following treatment with curcuminoids. AGS-BX1 cells were treated with several curcuminoids at their optimal lytic concentrations for 48 h. (**A**) The cells expressing GFP-lytic EBV reactivation were visualized under a fluorescence microscope at 20x magnification and the percentage of lytic cells was calculated. Approximately 3% of untreated AGS-BX1 cells expressed spontaneous lytic reactivation (DMSO); (**B**) Representative images (63× magnification) of GFP-positive HONE1-EBV cells induced by compound **EF24** compared to positive control GCb+VPA. For quantification, GFP-expressing EBV lytic cycle was classified into two category: low lytic (weak GFP expression) and high lytic (strong GFP expression); (**C**) The percentage of AGS-BX1 and HONE1-EBV cells induced into lytic cycle was estimated by calculating number of cells expressing low and high lytic activation. Although most cells induced by hit compounds expressed low lytic, strong GFP expressions (high lytic activation) were observed in approximately 20–22% of AGS-BX1 and HONE1-EBV cells upon **EF24** treatment.

Similar results were found for EBV lytic reactivation as visualized by fluorescence microscopy. Representative images of lytic induction by several curcuminoids in cell lines with recombinant EBV genomes are shown in Figure 3. In agreement with other studies [29,30,31], we found approximately 1–5% of untreated recombinant AGS-BX1 and HONE1-EBV cells to show spontaneous lytic reactivation (Figure 3A,B). To analyze the percentage of cells entering the lytic phase, we counted the number of cells expressing weak and strong green-fluorescence intensity and classified them into two categories; i.e., low and high EBV lytic reactivation (Figure 3B), as shown before [6]. Among curcuminoids, **EF24** induced EBV reactivation in AGS-BX1 cells at a comparable level to GCb+VPA (62–69%), whereas curcumin itself induced only 10% of AGS-BX1 cells into the lytic cycle (Figure 3A). Approximately 57% of HONE1-EBV cells exhibited GFP-expressing EBV lytic reactivation by **EF24** treatment. The lytic induction by GCb+VPA treatment was less in HONE1-EBV (45%, Figure 3C; right panel) as compared to AGS-BX1 cells (62%; Figure 3C; left panel). This indicates that cell background influences the permissiveness to induce EBV lytic reactivation. In addition, a higher level of spontaneous lytic reactivation of AGS-BX1 indicates that recombinant EBV in GC cells is more susceptible to chemical lytic inducing agents than recombinant EBV in NPC cells [6,12,24,28].

### 3.3. Curcuminoids Induce EBV Reactivation Better in Carcinoma Cells Carrying Recombinant EBV Compared to a Natural EBV Genome

**Figure 4:**
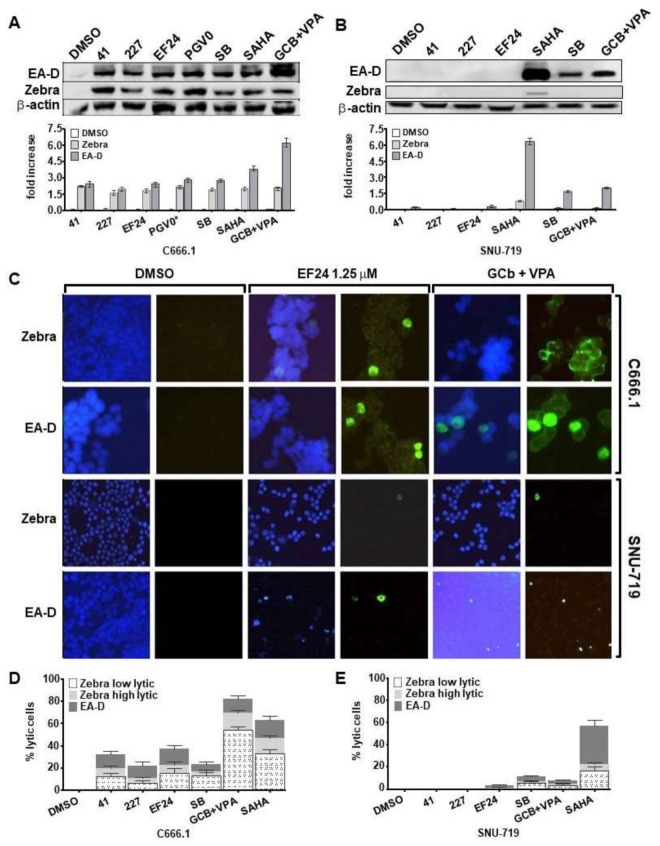
Curcuminoids have less lytic induction effects in natural EBV genome-carrying NPC and GC compare to recombinant carcinoma lines. Lead compounds at 1.25 µM lytic concentrations were able to induce EBV lytic cycle in natural EBV genome-carrying NPC (C666.1) but not GC (SNU-719) cells. The expression of Zebra and EA-D proteins upon 72–96 h treatment with curcuminoids analyzed by immunoblot and immunofluorescence staining. Proteins extracted from cells cultured with 5 µM SAHA, 3 mM SB, or 3 mmol/L GCb and 0.3 mmol/L VPA were loaded as positive controls. Cellular β-actin served as loading control. The induction of lytic EBV by curcuminoids is more effective in C666.1 (**A**) than SNU-719 cells (**B**); (**B**) SNU-719 expressed strong EA-D lytic proteins upon treatment with positive controls SAHA, SB and GCb+VPA; (**C**) Representative immunofluorescence images (40–63× magnification) of two EBV lytic cycle proteins in C666.1 and SNU-719 cells. Strong green fluorescence signals of Zebra and early antigen EA-D were identified upon **EF24** treatment in C666.1. Percentage of cells expressing Zebra and EA-D lytic proteins in C666.1 (**D**) and SNU-719 (**E**) cells. Zebra activation was detected in nearly 23% of C666.1 cells, whereas EA-D was expressed by approximately 14% of C666.1 cells upon EF24 treatment (**D**); Only SAHA induced significant Zebra and EA-D staining in SNU-719 cells. Approximately 70% of C666.1 (**D**) and 5% of SNU-719 (**E**) cells expressed EBV lytic proteins upon GCb+VPA treatment.

Next, we tested whether hit compounds (**41**, **227**, **EF24**) could induce EBV lytic cycle in natural EBV genome-carrying GC (SNU-719) and NPC (C666.1) tumor cell lines. We included GCb+VPA [6], SAHA [24,28] and SB [9,18] at optimal concentrations as positive controls. EBV lytic induction was achieved with all curcuminoid hit compounds in C666.1 giving higher (2.5–3 fold increase) Zebra and EA-D activation (Figure 4A) as compared to SNU-719 (Figure 4B). All three positive controls (SAHA, SB and GCb+VPA) induced strong EA-D activation in both cell types, whereas only SAHA induced Zebra and EA-D protein expressions in SNU-719 (Figure 4B). Curcuminoids with cyclopentanone linkers (e.g., **PGV0** 10 µM) induced EBV lytic activation in C666.1 NPC cells, but not in SNU-179 (data not shown). Compound **41** and **227**, which induced strong EBV lytic cycle in AGS-BX1 and HONE1-EBV, did not show lytic induction effects in SNU-719 (Figure 4B). Remarkably, of the curcuminoids only **EF24** promoted EBV lytic reactivation in SNU-719 cells (Figure 4C). Overall these data indicate differential EBV lytic induction effects for individual compounds when tested in a different cell background.

We further analyzed the percentage of C666.1 and SNU-719 cells induced into lytic cycle via immunofluorescent staining following treatment with 1.25 µM hit compounds or with the three positive controls. Representative immunofluorescence images of C666.1 and SNU-719 cells treated by **EF24** and GCb+VPA are shown in Figure 4C. As previously defined by Wildeman [6], the intensity of Zebra staining is categorized into low and high EBV level, whereas the EA-D staining was defined as a positive or negative signal, being a bright nuclear staining in all reactivating cells. Consistent with immunoblot results, we detected only limited expression of EA-D-positive SNU-719 cells induced by **EF24** (Figure 4C). On the contrary, in C666.1 NPC cells **EF24** induced the expression of Zebra (range from 15% to 20%) and EA-D (range from 12% to 18%) (Figure 4C,D). SAHA demonstrated strong lytic induction effects in up to 63% of C666.1 (Figure 4D) and in approximately 57% of SNU-719 cells (Figure 4E). Interestingly, treatment with GCb+VPA combination strongly induced the expression of Zebra-positive C666.1 cells up to 82%, whereas only approximately 12% of C666.1 cells expressed strong EA-D activation (Figure 4C,E), which is consistent to previous studies [6]. In contrast to the effects in C666.1, the CLVA drugs only induced weak (8%) EBV lytic activation in SNU-719 (Figure 4C,E). In summary, these data indicate that GC and NPC cells carrying recombinant EBV genomes, and particularly AGS-BX1 cells [12,28], are more inducible for EBV reactivation by curcuminoids compared to GC and NPC cells with natural EBV genomes. Furthermore, although **41** and **227** exhibit cell line-dependent induction, only **EF24** could induce EBV lytic cycle in all the EBV-positive carcinomas tested at micromolar concentrations. Of the three hit compounds, **227** showed the least lytic induction effect. Therefore, for further analysis, we focused on the curcuminoids **41** and **EF24**.

### 3.4. Analysis of Cell Viability at Different Concentrations of EBV Lytic Inducers

**Figure 5:**
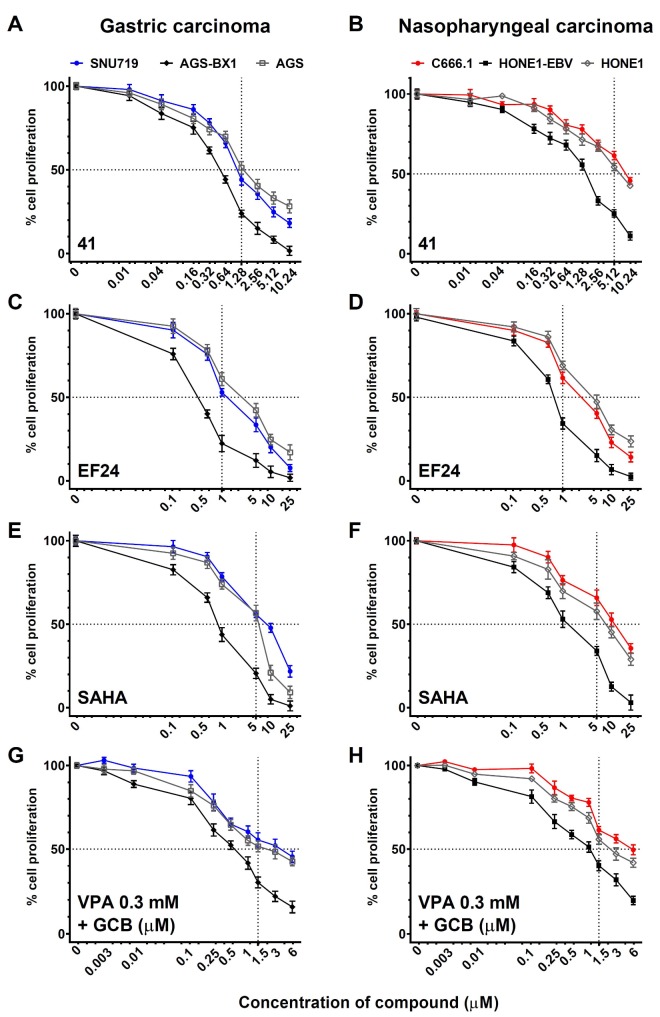
Naturally infected EBV positive carcinomas were more resistant to cytotoxicity effect of curcuminoids than those of recombinant EBV carcinoma cell lines. Cytotoxicity effect of hit compounds (**41**, **EF24**) was evaluated in GC (A,C,E,G) and NPC cells (B,D,F,H)). Treatments with SAHA and GCb+VPA were included as controls. AGS (EBV-negative), AGS-BX1 (recombinant EBV) and SNU-719 (native EBV) represent GC cells whereas HONE1 (EBV-negative), HONE1-EBV (recombinant EBV) and C666.1 (native EBV) represent NPC cells. All cells were incubated with various concentrations of lytic induction agents for 72 h and cell proliferation of treated cells was determined by 3-(4,5-dimethylthiazol-2-yl)-2,5-diphenyl tetrazolium bromide (MTT) assay. Results are expressed as percentages of treated cells compared with those of untreated cells and data from three independent experiments are presented. Standard deviation is shown in error bars. Dot line represents IC_50_ of compound/regimen in GC and NPC cells.

To examine the direct cytotoxic effect of **41** and **EF24** in GC and NPC cells, we performed MTT assays at 48 h post-treatment. Compound **41** and **EF24** demonstrated higher toxicity in GC (Figure 5A,C) and NPC (Figure 5B,D) cells containing recombinant EBV genomes (AGS-BX1 and HONE1-EBV) than the EBV-negative counterpart (AGS, HONE1, Figure 5A–D) at lytic induction concentration (1.25 µM), demonstrating EBV-specific effects of these curcuminoids. Compared to SNU-719 cells (Figure 5A,C), C666.1 cells treated by **41** and **EF24** demonstrated high lytic induction with less toxicity (Figure 5B,D). Two strong lytic induction agents, SAHA and GCb+VPA demonstrated cytotoxicity effects in a dose- and cell line-dependent manner (Figure 5E–H). Histone deacetylase inhibitor (SAHA) displayed higher toxicity than GCb+VPA in GC cell lines (Figure 5E), but SAHA was less toxic in NPC cell lines (Figure 5F), whereas GCb+VPA demonstrated minimal toxicity in both GC (Figure 5G) and NPC (Figure 5H) cell types.

Among lytic induction agents tested in this study, we found that **EF24** (Figure 5C,D) exhibited greater toxicity than SAHA (Figure 5E,F) even though its lytic inducing activity was lower than that of SAHA (Figure 4). This is consistent with previous studies on anti-proliferative activity of **EF24** against many types of cancer cells in vitro [18,21,22,23]. These findings suggest that the virus lytic inducing effect is not related to the cytotoxic potency of the compound. EBV-associated carcinoma cell lines containing recombinant EBV genomes (AGS-BX1, HONE1-EBV) are more prone to killing (Figure 5A,C,E), whereas native EBV genome-carrying C666.1 did not show virally-mediated killing at lytic inducing concentrations (Figure 5B,D,F). Taken together, we conclude that although EBV lytic cycle induced by curcuminoids is easily induced in cell lines artificially infected by EBV (AGS-BX1, HONE1-EBV), the authentic EBV-positive GC and NPC cell lines (SNU-719, C666.1) appear more resistant and may represent a more natural model to study viral reactivation in vitro.

### 3.5. Synergistic Effects of Curcuminoids in Combination with CLVA Regimen

**Figure 6:**
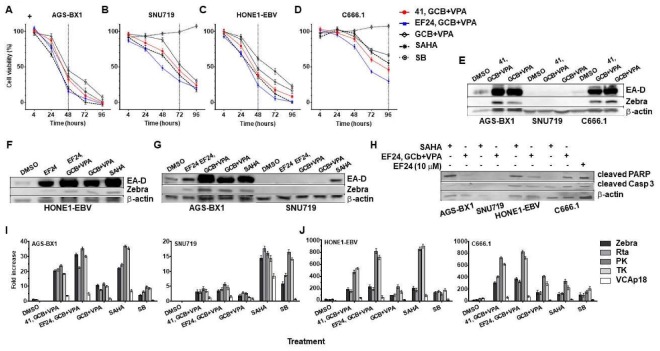
Combination of hit compounds and CLVA regimen synergistically induce EBV lytic cycle, significantly enhanced cell death and expressed apoptosis markers. Synergistic effects of hit compounds and CLVA regimen were measured by MTT assay, immunoblot and RT-qPCR quantification. EBV positive carcinomas: (**A**) AGS-BX1; (**B**) SNU-719; (**C**) HONE1-EBV; and (**D**) C666.1 cells were treated with hit compounds (**41, EF24**) in combination with GCb+VPA or GCb+VPA alone for 48–96 h followed by measurement of cell viability relative to untreated cells by MTT assay. Treatment with SAHA and SB were included as controls. Vertical dot line represents treatment time in each cell line; (**E**) AGS-BX1, SNU-719 and C666.1 cells were treated with a combination of **41** and GCb+VPA or GCb+VPA alone for 48–96 h and the expression of Zebra and EA-D lytic proteins were analyzed by immunoblot. Cellular β-actin served as loading control; (**F**) HONE1-EBV cells were treated with **EF24** and GCb+VPA, GCb+VPA alone or SAHA; (**G**). **EF24** significantly enhanced lytic induction capacity of CLVA regimen in EBV recombinant gastric carcinoma AGS-BX1, but not natural EBV genome-carrying SNU-719 cells. SAHA showed its capacity to induce EBV lytic in SNU-719. (**G**, right panel); (**H**) EBV-positive AGS-BX1, SNU-719, HONE1-EBV and C666.1 cells were treated with 5 µM SAHA (lane 1, 3, 5, 7) or **EF24** and GCb+VPA in combination (lane 2, 4, 6, 8) for 72 h followed by detection of expression of cleaved PARP and cleaved caspase-3 by immunoblot analysis. AGS-BX1 cells treated by 10 µM **EF24** was used as a control (lane 9) and cellular β-actin served as loading control. (**I**–**J**) RNA profiling of EBV-positive GC (**I**) and NPC (**J**) cell lines treated by either combination of hit compounds (**41, EF24**) with GCb+VPA or GCb+VPA alone. SAHA and SB treatments were used as positive controls whereas treatment with DMSO was used as negative control. RNA profiling of lytically induced-AGS-BX1 (**I**, left panel); -SNU-719 (**I**, right panel); -HONE1-EBV (**J**, left panel) and -C666.1 (**J**, right panel) cells showed increased levels of immediate early (Zebra and Rta) and early (PK, TK) lytic transcripts, whereas late lytic (VCA-p18) transcripts remained low or undetectable in all treated GC and NPC cells.

We previously reported that GCb+VPA can synergistically induce the EBV lytic in natural NPC cell lines [5,6,7,9]. Therefore, our next aim was to investigate a possible cooperative lytic induction effect of curcuminoids (**41**, **EF24**) and GCb+VPA in GC and NPC cells. As **41** and **EF24** significantly inhibit cell proliferation and reduce cell viability, we wonder whether these hit compounds in combination with GCb+VPA can enhance the cell killing. GC and NPC cells were treated with 1.25 µM concentration of hit compounds (**41**, **EF24**) and/or GCb+VPA. Treatment with SAHA and SB were included as positive controls in each cell line and cell viability was measured by MTT assay for 96 h. At 48–96 h post-treatment, **41** and **EF24** synergistically enhanced the cell killing effect of GCb+VPA in GC (Figure 6A,B) and NPC (Figure 6C,D) cells. Similar to cell killing effect of SAHA, these three compounds in combination (**41**, GCb+VPA; **EF24**, GCb+VPA) significantly reduce viability in AGS-BX1 (Figure 6A) and HONE1-EBV (Figure 6C) cells within 48 h, SNU-719 cells within 72 h (Figure 6B), and C666.1 cells within 96 h (Figure 6D). The synergistic cytotoxic effects of **EF24** combined with GCb+VPA in C666.1 cells was higher than SAHA (Figure 6D). In contrast to other lytic induction agents, SB induced tumor cell death only in GC and NPC cells carrying recombinant EBV genomes but not in carcinomas with native EBV, despite having the capacity to induce EBV reactivation in these cells (Figure 4A,B). Our data indicate that curcuminoids can enhance cell death when combined with GCb+VPA in EBV-associated carcinomas.

To determine whether curcuminoids can improve induction of lytic EBV and enhance the killing effect of the CLVA regimen (GCb+VPA), the EBV lytic protein and lytic mRNA expression were examined by immunoblot and real-time quantitative RT-qPCR [6,30,32] in EBV-positive GC and NPC cells treated with **41** or **EF24** given as adjuvant to CLVA treatment. We found that although **41** could cooperate with GCb+VPA, both demonstrated lytic reactivation on their own (Figure 2G and Figure 4A,D) and **41** did not enhance the activation of EBV lytic proteins (Figure 6E). On the other hand, combining **EF24** with GCb+VPA not only caused enhanced cell death (Figure 6A–D) but also significantly enhanced the expression of EBV lytic proteins in both EBV-positive GC and NPC cells (Figure 6F,G, Supplementary Materials Figure S2).

We also examined the effect of pre-treatment with curcuminoids prior to GCb+VPA on EBV reactivation in recombinant AGS-BX1 cells. Following pre-treatment for 24 h and a further 24 h GCb+VPA incubation, cell extracts were collected for immunoblot analysis. Surprisingly, the induction of EBV lytic proteins (Zebra and EA-D) in response to CLVA (GCb+VPA) treatment was downregulated by pre-treatment with curcuminoids for 24 h (Supplementary Materials Figure S3). Curcuminoids, which activated EBV in C666.1 (Figure 6E) HONE1-EBV (Figure 6F), and AGS-BX1 (Figure 6E,G) cells, synergized with CLVA regimen when used as adjuvant and added simultaneously. Taken together, our data suggest that the activation of EBV lytic cycle by curcuminoids is independent of their cytotoxic potency and simultaneous administration of curcuminoids with CLVA regimen provides the best lytic induction.

### 3.6. Effect of Combined EF24 and CLVA Treatment on Apoptosis and EBV Lytic Protein Expression in GC and NPC Cells Carrying Artificial EBV Genomes

The variation of curcuminoids in activating EBV lytic cycle in different cell types indicates possible mechanistic differences in cell line-dependent lytic reactivation. As replication of EBV can be triggered by apoptosis [33], we conducted experiments to investigate the effect of curcuminoids and SAHA on cell apoptosis. The expression levels of proteolytic cleavage of caspase-3 and PARP, both markers of cell apoptosis were analyzed by immunoblot. To accomplish this, EBV-positive GC and NPC cells were treated with combined **EF24** and CLVA regimen. SAHA treatment was included as additional lytic induction control [24,28]. Since the cytotoxic effect of many drugs is greater in AGS-BX1 compared to other cell lines (Figure 5 and Figure 6A), we used AGS-BX1 treated with 10 µM **EF24** to identify increased levels of both apoptotic markers [12,28].

Effective induction of the EBV lytic cycle in HONE1-EBV (Figure 6F) and AGS-BX1 (Figure 6G; left panel) cells was confirmed by the expression of Zebra and EA-D lytic proteins following combined **EF24**, GCb+VPA or SAHA treatments. AGS-BX1 and HONE1-EBV cells treated with combined **EF24**, GCb+VPA did not show increased levels of both cleaved caspase-3 and PARP proteins. SAHA treatment showed a tendency to increase cleaved PARP protein levels in recombinant GC and NPC cell lines (Figure 6H). In contrast to AGS-BX1 cells, cleaved PARP protein levels were enhanced in C666.1 cells by combined **EF24**, GCb+VPA treatment but not by SAHA. The relation between EBV lytic protein expression (Figure 6E) and apoptosis of EBV-positive cells was more clearly observed in C666.1 cells (Figure 6H). Interestingly, SNU-719 cells which relatively sensitive to cytotoxic effect of combined **EF24**, GCb+VPA treatment (Figure 6B) but resistance to EBV lytic reactivation (Figure 6G; right panel) did not express either cleaved caspase-3 or PARP protein markers (Figure 6H). Taken together, our data indicate that apoptosis induction is not linked to EBV lytic activation and vise versa. In agreement with previous studies [21,31,33], we observed apoptosis-associated EBV lytic reactivation to be more prominent in GC and NPC cells transformed by recombinant rather than in those carrying native EBV genomes. It is possible that the apoptotic pathways in naturally EBV infected cell lines exhibit important differences compared to EBV recombinant cell lines.

### 3.7. EBV Immediate-Early and Early Rather Than Late Lytic Gene Expression Is Induced by Combination Curcuminoid and CLVA Regimen Treatment in EBV-Positive Carcinomas

Previous studies indicated that the combination of GCb+VPA could increase EBV RNA levels encoding Zebra, protein kinase (PK), thymidine kinase (TK), and small capsid protein (VCA-p18) in AGS-BX1 and C666.1 cells [6,27]. Our data show that the combination of curcuminoids (**41**, **EF24**) with GCb+VPA resulted in a slight increase in immediate early (ZEBRA) and early (EA-D) protein levels in both the EBV-positive GC and NPC cells (Figure 6E–G). To determine whether a combination of curcuminoid with GCb+VPA treatment activates EBV lytic genes, we performed qRT-PCR (24) from EBV-positive GC and NPC cells that had been exposed to a combination of CLVA regimen and **41** or **EF24**. SAHA and SB treatments were included as additional controls.

In AGS-BX1 cells, curcuminoid **EF24** together with GCb+VPA increased mRNA levels relative to DMSO of immediate-early (Zebra ~30-fold; Rta ~20-fold), early (PK ~35-fold; TK ~30-fold), and late lytic mRNAs (VCAp18 ~5-fold) 48 h after treatment, whereas a treatment with GCb+VPA alone increased the EBV lytic gene expression approximately 10-fold (Figure 6I). DMSO alone had little effect in all cells (Figure 6I,J) except SNU-719. When EBV-positive NPC cells (HONE1-EBV, C666.1) treated with **41** or **EF24** for 48 h together with the CLVA regimen, EBV lytic gene activation was increased even higher. Zebra mRNA activation abundance was ~200- to 370-fold, Rta was ~200- to 410-fold, PK was ~480- to 830-fold, TK was ~540- to 720-fold, and VCAp18 was ~54- to 77-fold (Figure 6J). A combination of **EF24** with GCb+VPA induce higher EBV mRNA levels than **41** with GCb+VPA in all cells except SNU-719 (Figure 6I). SAHA treatment induced similar EBV mRNA levels as **EF24** with GCb+VPA. Our data indicate that use of curcuminoid(s) as adjuvant for CLVA treatment increases EBV reactivation at the mRNA level above GCb+VPA alone (Figure 6I,J) and the lytic induction effect of this drug combination (EF24 with GCb+VPA) is similar to SAHA. Taken together, curcuminoid **EF24** has the most potent in vitro EBV inducing activity and could acts as adjuvant for CLVA therapy.

## 4. Discussion

EBV is consistently present in every tumor cell of EBV-positive cancers in a state of latency characterized by a restricted gene expression [1,2,4,9]. Despite generally good responses to standard chemoradiation therapy at early tumor stage recurrences and disease progression are common and alternative (virus targeted) therapies are needed [2,3,4,5]. Strategies for targeting the virus have been explored, including the concept of reactivating latent viral genomes in combination with antiviral therapy [5,6,7,8,9]. Anti-herpes viral drugs that rely on phosphorylation by viral thymidine kinase for conversion of the prodrug to its active form, are not effective during latent infection [5,9]. Several drugs such as 5-azacitidine (5-Aza) [9], GCb+VPA [6,7] and SAHA [24,28] have been used in combination with antiviral agents in clinical studies, however, it remains unclear if the response observed was truly related to lytic induction and antiviral drug (ganciclovir) sensitization of tumor cells. Thus, discovering new and toxicity-limiting EBV lytic inducing agents to boost viral reactivation and selective elimination of EBV-carrying cells merits investigation [11,12].

The possibility of using a dietary constituent such as curcumin to induce lytic cycle in latently-infected EBV cells has remained largely unexplored [13,14]. Novel curcuminoids with enhanced solubility and oral availability, improved safety and/or minimal side effects [15,16,17,18,19,20,21,22,23] offer the option as a supportive oral approach to the current CLVA therapy, either as single agents or in combination with other lytic inducers. The present study was conceptualized to determine the effects of curcuminoids on the induction of EBV lytic cycle. We demonstrated that lead compounds **41**, **227** and **EF24** could effectively induce the EBV lytic reactivation in vitro. Importantly, we showed that **41** and **EF24** synergistically induced stronger activation of EBV lytic cycle in GCb+VPA treated EBV-positive GC and NPC lines. Tumor cell viability significantly decreased, in particular by the combination of GCb+VPA and the piperidinone **EF24**. Taken together, our data suggest that the activation of EBV lytic cycle by curcuminoids may provide a novel adjuvant approach for targeted treatment of EBV-associated gastric and nasopharyngeal carcinomas.

Most existing lytic inducing agents are frequently cell line specific or reactivate a limited number of cells [6,8,9,10,11,12,30]. Here, we observed a remarkable increase in EBV lytic reactivation and concurrent reduction in cell proliferation leading to apoptosis in cells containing recombinant EBV genomes (AGS-BX1, HONE1-EBV) upon treatment with lead compounds **41**, **227** and **EF24**. Similar effects were observed in natural EBV genome-carrying NPC cell line (C666.1), but the effects were less pronounced in the natural genome-carrying GC cell line SNU-719. Compounds **41** and **EF24** did have anti-proliferative activity in SNU-719 cells and enhanced anti-proliferative activity of GCb+VPA. Furthermore, we observed that the anti-proliferative activity of these compounds was not EBV-specific, confirming prior studies in other cancer models [13,14,16,17,18,19,20,33,34]. Hence, curcuminoids may have lytic inducing capacity to trigger EBV reactivation from latency independent of their anti-proliferative activities. GC and NPC lines induced for lytic EBV gene expression are therefore more sensitive to killing by the antiviral agent valGCV.

The mechanisms that limit the level and speed of virus reactivation in different cell types or between cells in the same culture population are not well understood [10,11,32,34], but possibly relate to differences in histone modification and promotor methylation of host and viral genes [2,5,6]. The EBV-genome in recombinant cell lines may be more susceptible to virus reactivation, due to limited methylation of host and virus genomes [2,5,8,9,10,11,12,35,36,37]. In cell lines carrying a natural EBV genome, like the C666.1 and SNU-719 used here [6,20,37], the viral genome may be more reluctant to reactivation signals, and perhaps be considered as the more appropriate model systems [35,36]. In agreement with others [24,28], our study showed that SAHA is a potent lytic inducing agent in all types of EBV-positive cells, including NPC and GC cell lines (C666.1, SNU-719) harbouring native EBV genome [9,24,28]. Although the C666.1 prototype NPC cell line can be readily induced for EBV reactivation, the prototype EBVaGC line SNU-719 appears least sensitive to lytic induction by curcuminoids. We conclude that host cell-specific factors (i.e., extensive genome methylation and histone modification) are contributing to the latency program in SNU-719 cells and affect EBV lytic reactivation following curcuminoid treatment [2,37,38].

Recently, we showed that GCb+VPA can trigger EBV lytic replication in NPC patients in vivo creating therapeutic sensitivity to antiviral treatment [5,6,7]. However, this treatment still needs improvement. Therefore we investigated the possible synergistic effects of combinations of curcuminoids (**41**, **EF24**) with GCb+VPA in various EBV-linked carcinoma model cell lines. We observed that the combination of **EF24** and GCb+VPA most effectively enhanced the reactivation of EBV lytic cycle in EBV-associated carcinoma cell lines, with the exception of SNU-719 cells. Reactivation was accompanied by a reduction of tumor cell viability. SAHA proved more broadly effective in EBV reactivation and triggered EBV reactivation even in the SNU-719 cell line. The synergistic induction of EBV lytic cycle combined with enhanced cell death in NPC and GC cells carrying recombinant EBV genomes, reflect the enhanced susceptibility of the recombinant lines to lytic induction.

It is well accepted that lytic activation in itself may render the infected cells more susceptible to immune recognition and killing due to the expression of viral lytic-switch proteins, in particular Zebra [4,8,9,30,31]. Interestingly, while the curcuminoids promoted EBV lytic induction by GCb+VPA when added simultaneously, they showed inhibitory activity against EBV reactivation when added separately prior to GCb+VPA treatment (supplementary Materials Figure S3). These findings support other studies showing that, apart from their potential as viral lytic activators [9,38], natural dietary compounds such as resveratrol, sulfronane and luteolin [39,40,41] inhibit the EBV lytic cycle when added prior to viral reactivation by chemical treatment [9]. Simultaneous addition of enhancing agents provides a synergy effect on viral reactivation. RNA profiling revealed that a combination of a curcuminoid (**41** or **EF24**) with GCb+VPA induced immediate-early and early lytic rather than late lytic gene expression. In agreement with previous publications, low or undetectable levels of full EBV late lytic gene expression upon EBV reactivation by chemical treatment are suggestive of abortive lytic replication [24,30,32,42]. However, even abortive reactivation will induce immune sensitization by expression of (immediate) early gene products [5,6,7]. 

Taken together, the combination of a curcuminoid, in particular compound **EF24** with the CLVA regimen at clinically acceptable doses, may enhance EBV reactivation and provide an attractive and safe therapeutic strategy for EBV-associated malignancies. A similar beneficial effect may be expected when using SAHA (Vorinostat) or the recently identified HDAC inhibitor romidepsin in the CLVA treatment regimen [43]. Combined use of oral curcuminoids and SAHA or romidepsin may provide synergistic effects, because each agent triggers EBV reactivation by a different mechanism. Further studies are needed to substantiate this is across several EBV carrying cell lines and tumor models. With the advent of oral formulations of gemcitabine [44,45] and oral valGCV, a fully oral CLVA treatment option may become available for virus-targeted therapy of EBV-associated cancers, which will be of particular benefit for developing countries where radiotherapy and intensive care options are limited [46].

## 5. Conclusions

In conclusion, our study identified two curcuminoids bearing the thiopyranone dioxide (**41**) and piperidinone (**EF24**) scaffolds that serve as putative EBV lytic activators in latently infected EBV-positive carcinomas. **EF24** has the potential to act as an adjuvant to enhance EBV reactivation induced by the CLVA regimen (GCb+VPA) for treatment of EBV-associated gastric and nasopharyngeal carcinomas.

## Supplementary Materials

The Supplementary materials are available online at http://www.mdpi.com/2072-6694/10/4/89/s1. Figure S1: Curcuminoids induced weak EBV lytic reactivation at low (10 nM) concentrations, Figure S2: Representative immunofluorescence images of C666.1 cells treated by hit compounds (**41**, **EF24**) alone or in combination with GCb+VPA. (A). C666.1 expressed Zebra and EA-D lytic proteins upon **EF24** treatment alone or in combination with GCb+VPA. (B). C666.1 expressed Zebra and EA-D lytic proteins upon **41** treatment alone or in combination with GCb+VPA. Increased number of cells expressing Zebra and EA-D lytic proteins upon co-treatment with **EF24**, GCb+VPA (left panel) or with **41**, GCb+VPA (right panel) were shown, Figure S3: Pre-treatment with curcuminoid inhibits EBV lytic cycle induced by CLVA regimen. C666.1 cells were treated with hit compounds (**41**, **EF24**) 24 h before and after GCb+VPA administration. The cells were collected after 96 h followed by immunoblot detection of Zebra and EA-D proteins. Treatment with hit compounds or GCb+VPA alone for 96 h were included for comparison. When administered simultaneously with CLVA regimen, hit compounds (**41**, **EF24**) synergistically induced EBV reactivation. Curcuminoids antagonized the lytic induction effect of GCb+VPA when administered 24 h prior GCb+VPA treatment.
