# Supplementary material for: Curcuminoids as EBV Lytic Activators for Adjuvant Treatment in EBV-Positive Carcinomas
Source: Cancers (Basel). 2018 Mar 22;10(4):89. doi: 10.3390/cancers10040089 (PMC5923344; doi:10.3390/cancers10040089)
Supplement: Supplementary file 1 [file cancers-10-00089-s001.pdf]

Supplementary Materials: Curcuminoids as EBV lytic activators for adjuvant treatment in EBV-positive carcinomas

Octavia Ramayanti, Mitch Brinkkemper, Sandra A.W.M Verkuijlen, Leni Ritmaleni, Mei Lin Go, Jaap M. Middeldorp

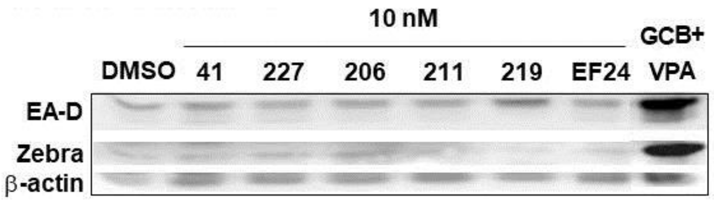

Figure S1. Curcuminoids induced weak EBV lytic reactivation at low (10 nM) concentrations.

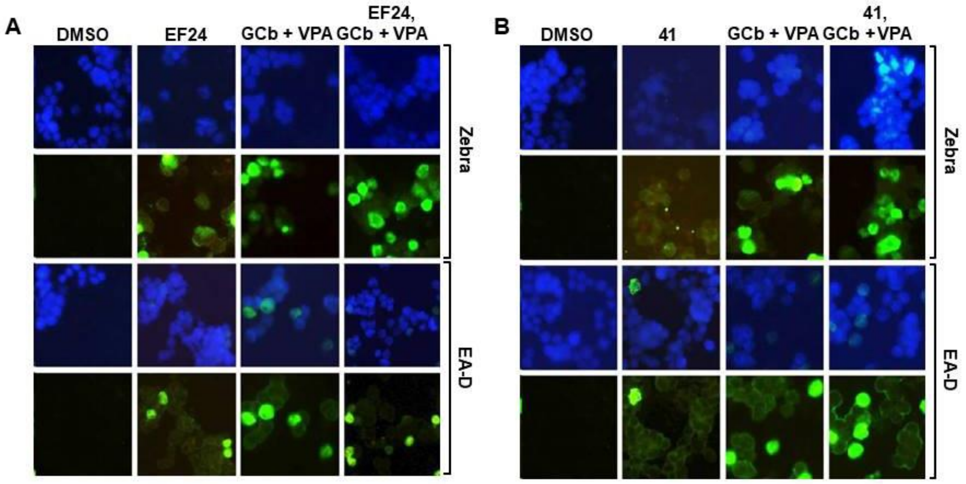

Figure S2. Representative immunofluorescence images of C666.1 cells treated by hit compounds (41, EF24) alone or in combination with GCb+VPA. (A). C666.1 expressed Zebra and EA-D lytic proteins upon **EF24** treatment alone or in combination with GCb+VPA. (B). C666.1 expressed Zebra and EA-D lytic proteins upon **41** treatment alone or in combination with GCb+VPA. Increased number of cells expressing Zebra and EA-D lytic proteins upon co-treatment with **EF24**, GCb+VPA (left panel) or with **41**, GCb+VPA (right panel) were shown.

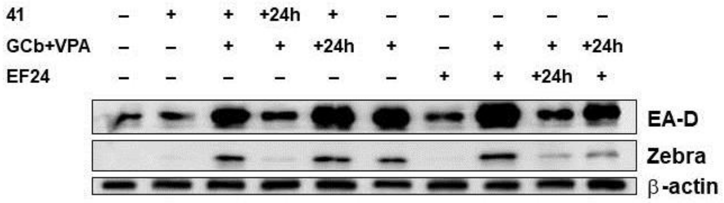

Figure S3. Pre-treatment with curcuminoid inhibits EBV lytic cycle induced by CLVA regimen. C666.1 cells were treated with hit compounds (**41**, **EF24**) 24 h before and after GCb+VPA administration. The cells were collected after 96 h followed by immunoblot detection of Zebra and EA-D proteins. Treatment with hit compounds or GCb+VPA alone for 96 h were included for comparison. When administered simultanously with CLVA regimen, hit compounds (**41**, **EF24**) sinergistically induced EBV reactivation. Curcuminoid antagonized the lytic induction effect of GCb+VPA when administered 24 h prior GCb+VPA treatment.

© 2018 by the authors; licensee MDPI, Basel, Switzerland. This article is an open access article distributed under the terms and conditions of the Creative Commons by Attribution (CC-BY) license (http://creativecommons.org/licenses/by/4.0/)
